# Depressed mood affects the process of biological aging, analyses from the NHANES dataset

**DOI:** 10.3389/fragi.2025.1516664

**Published:** 2025-07-08

**Authors:** Yuan Tian, Qiao Lu, Jing Li, Xiaobo Zhou, Luyao Wang, Xuemei Zhong, Yiping Luo

**Affiliations:** ^1^ Sichuan Provincial Center for Mental Health, Sichuan Provincial People’s Hospital, School of Medicine, University of Electronic Science and Technology of China, Chengdu, China; ^2^ Key Laboratory of Psychosomatic Medicine, Chinese Academy of Medical Sciences, Chengdu, China

**Keywords:** depressed mood, biological aging, aging, actual age, NHANES

## Abstract

**Background:**

Depressive mood may influence biological aging and the difference (δ-age) between biological age (BA) and chronological age (CA). This study explores the relationship between depressive mood and whole-body delta age (δ-age).

**Methods:**

A total of 7,383 U.S. adults were selected from the National Health and Nutrition Examination Survey (NHANES) conducted between 2007 and 2018. Depressed mood was evaluated using PHQ-9 scores. Biological age (BA) was estimated based on circulating biomarkers, and the calculated delta age (δ-age) was validated through a generalized linear regression analysis.

**Results:**

After adjusting for confounding variables, logistic regression analysis demonstrated a significant association between elevated depressive symptoms and accelerated biological aging. The restricted cubic splines (RCS) results further indicated a positive dose-response relationship between depression scale scores and the risk of biological aging. Additionally, the weighted quantile sum regression (WQS) findings revealed a positive, though non-significant, trend linking depressive mood to the risk of biological aging. Notably, overeating and low self-perception emerged as the most significant contributors to the scores on the Patient Health Questionnaire-9 (PHQ-9) scale.

**Conclusion:**

Depressive symptoms are linked to accelerated biological aging. Thus, interventions aimed at improving mood may help slow biological aging and contribute to delaying the aging process.

## 1 Introduction

With advances in medical technology, average life expectancy has gradually increased, from 60.8 years in 2005 to 70.8 years in 2015(Source: United Nations Statistical Yearbook, 2017 edition. Data refers to a 5-year period preceding the reference year). However, the rise in average age has been accompanied by an increased prevalence of age-related diseases, thereby imposing a growing burden on societal and healthcare systems (Ye et al., 2023). Aging is characterized by degenerative changes in the structural integrity and physiological functions of body tissues. Additionally, individual variability plays a significant role in the manifestation and progression of age-related changes. The progression of aging is not uniform across individuals, inability to accurately mirror the patient’s actual condition. So, aging is commonly quantified by BA. Aging, also referred to as biological aging (Dodig et al., 2019), was influenced by a wide range of elements, including environmental aspects, genetics, lifestyle choices, and psychological wellbeing. BA is proposed as a metric to assess the aging state of the human body. BA more accurately reflects the physiological aging process than CA (Kwon and Belsky, 2021). BA is derived from the assessment of normal human physiological and anatomical development, can indicate the current state of the body’s organizational and physiological structures. BA can represent inflammatory signaling from genomically damaged or senescent cells. Telomere dysfunction can initiate and maintain inflammation on several levels. Therefore, BA not only serves as a biomarker of aging, but also reflects deteriorations in multiple health parameters including immune competence, reproductive capacity, cardiac function, and increased cancer susceptibility (Chakravarti et al., 2021). BA is calculated using a comprehensive set of parameters, including hematological indicators and DNA methylation (Hamczyk et al., 2020). The pursuit of robust depression-aging biomarkers demands integrative frameworks reconciling technical constraints and cost-efficacy. DNA methylation, while predominant, requires synergistic coupling with physiological indices through multi-omics pipelines. This paradigm shift emphasizes cross-platform biomarker convergence, leveraging machine learning algorithms to optimize cost-performance ratios while maintaining clinical interpretability. Lifestyle factors, such as smoking, excessive alcohol consumption, and chronic sleep deprivation, are associated with accelerated aging, consequently elevating BA (Friedman, 2020). Physical health constitutes a significant component of overall wellbeing and is intricately linked to disease susceptibility. Anxiety disorders are associated with higher rates of cardiovascular, respiratory, and gastrointestinal disorders (Meuret et al., 2020). Major depression is a high-risk factor for suicide (Lundberg et al., 2023). Current research links depression and aging with heightened oxidative stress and a concurrent inflammatory response (Ji et al., 2023). Depression and aging can diminish the health status of older adult individuals. Despite the growing interest in the intersection of mental health and physiological processes, a few studies to date have specifically examined the relationship between depression and biological age. Clinical studies have documented accelerated biological aging in major depressive disorder (MDD), primarily through epigenetic biomarkers (e.g., DNA methylation patterns) and neuroanatomical indicators. Severe depression shows significant correlations with elevated epigenetic aging, as measured by DNA methylation (DNAm) changes, where depressive symptoms independently predict age-associated acceleration of DNAm alterations. Machine learning models additionally detect increased brain age gaps between MDD patients and healthy controls. These findings characterize depression as a modifiable risk factor for accelerated aging, suggesting mood regulation could serve as a practical target for anti-aging interventions. Consequently, targeted modulation of emotional states might decelerate biological aging processes, thereby reducing the growing burden on healthcare and older adult care systems caused by population aging (Lorenzo et al., 2023). The National Health and Nutrition Examination Survey (NHANES) database is a large continuous cross-sectional sample database in the United States. We will use data from 6 cycles from 2007 to 2008 to 2017–2018 for our analysis, the association was substantiated by quantifying BA, denoted as δ-age (δage = BA-CA), alongside DPQ scores.

## 2 Materials and methods

### 2.1 Study population

**FIGURE 1:**
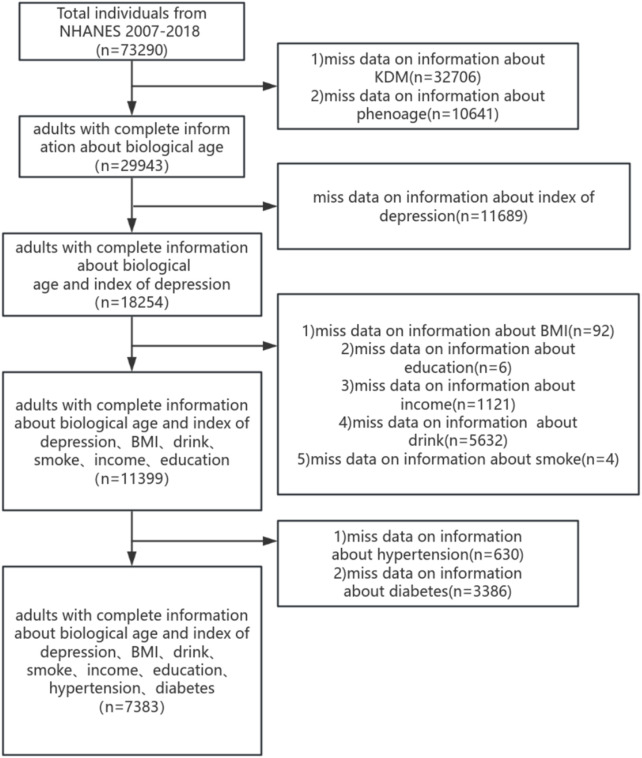
Flowchart portraying the sample selection.

The NHANES database is an annual cross-section conducted in the United States each year, including a health interview survey and physical health survey of participants. All collected from NHANES participants have been approved by the NCHS Ethics Review Board. (Available online:https://www.cdc.gov/nchs/nhanes/). In this study, we analyzed the depressed mood scores of 7,383 participants across NHANES cycles spanning 2007–2008 to 2017–2018. Subjects under the age of 20 and those incapable of undergoing BA assessment or completing depression scale scoring were excluded from the study. Collecting participant information on demographics, health-related lifestyles and chronic diseases, a final total of 7,383 individuals were included to participate in this study, and the process is shown in Figure 1.

### 2.2 Depressed mood

The PHQ-9 scale, also recognized as the patient health questionnaire, serves as a depression screening instrument. The PHQ-9 was utilized to assess the frequency of depressive symptoms and the impact on social functioning over the preceding 2-week period. The scale contains 10 questions, score each question 0–3. The PHQ-9 was employed to evaluate the frequency of depressive symptoms and their influence on social functioning within the last 2 weeks. Assessment with the PHQ-9 scale adheres to the DSM-IV diagnostic criteria for depression (Liu et al., 2023).

### 2.3 Biological aging

To calculate BA. The NhanesR package developed by Jing Zhang in R Studio was used, as used by Jianmin Zhu et al. The study determined the BA across various systems, encompassing whole-body, cardiovascular, renal, and hepatic bioages. Systemic BA was calculated utilizing the mean values of diastolic and systolic blood pressures, total cholesterol, alkaline phosphatase, creatinine, uric acid, total leukocyte count, lymphocyte count, hemoglobin, HbA1c, mean cell volume, and C-reactive protein. Ultimately, the δ-age for each BA assessment was determined by subtracting the individual’s CA from their BA (δ-age = BA- CA) (Kwon and Belsky, 2021). We will employ two types of BA to better facilitate our assessment. The main formula for calculating BA is as follows: KDM (Klemera-Doubal Method) = [Σ ((B_i - B̂_i(a))/σ_Bi) × (∂B̂_i(a)/∂a)]/[Σ (∂B̂_i(a)/∂a)^2^] (Klemera and Doubal, 2006). PhenoAge (Phenotypic Age):1,S = 0.007 × Albumin (g/dL)−0.009 × Creatinine (mg/dL) + 0.027 × Glucose (mg/dL) + 0.014 × log (CRP) (mg/L) + 0.013 × Lymphocyte (%) + 0.007 × MeanCellVolume (fL) + 0.027 × RDW (%) + 0.053 × ALP(U/L) + 0.005 × WBC(×10^9^/L) + 0.079 × ChronologicalAge (years). 2,PhenoAge = 141.50 + [ln (−ln (0.5)/0.0076927 × S]/0.0076927 (Levine et al., 2018).

### 2.4 Variables

A suite of general variables was employed in the statistical analysis of this study. In this study, statistical analysis encompassed age, gender, race, smoking status, alcohol consumption, BMI, and the presence of multiple chronic conditions.

Participants were stratified into three distinct age groups: under 40 years group, 40–59 years group, and 60 years + group (Yan et al., 2022). Race was categorized as Hispanic group, Non-Hispanic White group, Non-Hispanic Black group and other Race (Chen et al., 2022). Income was categorized as 0∼130 FPL group, >130∼350 FPL group, >350 FPL group (Ruan et al., 2022). Overweight individuals were identified as those with a BMI ≥25 kg/m^2(^(Kim et al., 2022)^)^. Alcohol users were categorized into the following groups: “non-drinkers”, “former drinkers”, “light drinkers”, “moderate drinkers” and “heavy drinkers” (Rattan et al., 2022). Smoking status was defined as previous or current smoking (Nie et al., 2023). Physical activity was measured in Metabolic Equivalent (MET) minutes per week (Ainsworth et al., 1993). Hypertension was defined based on blood pressure measurements and self-report (Cao et al., 2023). Diabetes was defined based on fasting blood glucose, glycated hemoglobin (HbA1c), oral glucose tolerance test (OGTT), and self-report (Chen et al., 2023).

### 2.5 Statistical analysis

NHANES-suggested weights are used to account for planned oversampling of specific groups. Continuous variables are expressed as mean ± standard deviation, while categorical variables are expressed as counts (percentages). Then, χ2 was tested and a one-way analysis of variance (ANOVA) was performed to assess the association between the independent variables, each BA and each δ-age. Furthermore, generalized linear regression assessed the association between depressive mood and δ-age for each participant. Also adjusting for covariates, the RCS was used to test for dose-response relationships. The WQS model was used for mixed effects analysis. All statistical analyses were conducted utilizing R software (version 4.1.2), RStudio software and the rcs and gwqs software packages. The models were adjusted for covariates including age, sex, race/ethnicity, educational attainment, hypertension, diabetes mellitus, smoking status, physical activity levels, and other relevant variables. The node selection in the restricted cubic spline (RCS) models was implemented using the rms package (version 6.7–0), with three knots specified as the default configuration. We assessed normality with QQ plots and density plots aligned to a normal distribution.

## 3 Results

### 3.1 Basic clinical characteristics of study participants

A total of 7,383 participants were enrolled in this study. The clinical characteristics of the study population for each independent variable are presented in Table 1. The distribution of depressed mood and age across tertiles is detailed in Table 1. The mean age of the study population was 44.71 years, with a standard deviation of 16.00 years. Biological age-accelerated aging is evident across all age groups, with depression prevalence demonstrating variability among these groups. Males accounted for 3,597 cases (46.6%) and females for 3,786 cases (53.4%). The majority of participants were Hispanic Americans (71.4%). Most have a high school diploma or higher (65.1%) and mostly at higher income levels (46.5%). Individuals who rarely consume alcohol may still be prone to high blood pressure, while those who smoke are at an increased risk of being overweight, defined as having a BMI of 25 or higher.

**TABLE 1:** Characteristics of NHANES participants by tertiles of depressed mood scores.

Characteristics (weighted%)	Overall	20–40 y	40–60 y	60 + y	*P*
n	7,383	2,971	2,575	1837	
Gender					
Male	3,786 (53.4)	1,554 (51.6)	1,328 (52.3)	904 (59.1)	<0.001
Female	3,597 (46.6)	1,447 (48.4)	1,246 (47.7)	933 (40.9)	
Race					<0.001
Hispanic	3,489 (71.4)	1,269 (63.6)	1,177 (73.0)	1,043 (84.8)	
Non-hispanic white	1,359 (9.3)	550 (10.7)	504 (9.5)	305 (6.0)	
Non-hispanic black	1,119 (7.7)	511 (10.8)	397 (6.7)	211 (3.1)	
Other race	1,416 (11.5)	641 (14.9)	497 (10.8)	278 (6.1)	
Education					0.332
< High school	1,452 (12.0)	516 (12.0)	523 (12.0)	413 (11.8)	
High school/GED	1707 (22.9)	661 (21.7)	591 (22.8)	455 (25.6)	
College or above	4,224 (65.1)	1794 (66.4)	1,461 (65.1)	969 (62.6)	
Family income					<0.001
0∼130 FPL	2091 (18.7)	972 (24.3)	697 (15.6)	422 (13.0)	
>130∼350 FPL	2,847 (34.8)	1,200 (39.0)	892 (29.3)	755 (36.6)	
>350 FPL	2,445 (46.5)	799 (36.7)	986 (55.1)	660 (50.4)	
BMI (mean (SD))	29.29 (7.00)	28.67 (7.48)	29.82 (6.75)	29.55 (6.32)	<0.001
Smoke					
no	3,719 (48.6)	1,314 (44.6)	1,326 (49.1)	1,079 (55.9)	<0.001
yes	3,664 (51.4)	1,657 (55.4)	1,249 (50.9)	758 (44.1)	
Drink (mean (SD))	0.02 (0.14)	0.03 (0.17)	0.02 (0.13)	0.00 (0.05)	<0.001
PA (mean (SD))	625.32 (701.06)	671.43 (540.66)	568.61 (575.14)	638.06 (1,098.04)	<0.001
Diabetes (%)					
no	1,075 (10.7)	106 (3.2)	428 (12.8)	541 (22.6)	<0.001
yes	6,308 (89.3)	2,865 (96.8)	900 (87.2)	1,296 (77.4)	
Hypertension					
no	3,300 (40.3)	588 (19.7)	1,293 (46.3)	1,419 (71.8)	<0.001
yes	4,083 (59.7)	2,383 (80.3)	1,282 (53.7)	418 (28.2)	
Age (mean (SD))	44.71 (16.00)	29.11 (5.65)	49.25 (5.66)	68.52 (6.32)	<0.001
phenoage (mean (SD))	43.67 (17.44)	27.79 (7.93)	48.19 (8.37)	68.14 (9.63)	<0.001
phenoage_advance (mean (SD))	−1.04 (5.96)	−1.32 (5.32)	−1.07 (6.22)	−0.39 (6.63)	0.004
kdm (mean (SD))	32.91 (14.47)	22.46 (8.97)	36.53 (11.19)	47.76 (12.90)	<0.001
kdm_advance (mean (SD))	−11.80 (11.58)	−6.65 (8.61)	−12.72 (10.57)	−20.76 (12.87)	<0.001
DPQ (mean (SD))	4.64 (4.44)	4.68 (4.35)	4.91 (4.77)	4.04 (3.90)	<0.001
Depression					0.001
no	6,347 (87.9)	2,539 (87.1)	2,163 (86.7)	1,645 (91.7)	
yes	1,036 (12.1)	432 (12.9)	412 (13.3)	192 (8.3)	

### 3.2 Study of the difference between biological and actual age in the population

Table 1 shows the mean and standard deviation values of BA and δ-age for each categorical variable. BA (PHE) for those less than or equal to 40 years of age was 27.79 ± 7.93, with a δ-age of −1.32 ± 5.32. BA for 40–60 year olds 48.19 ± 8.37, δ-age is −1.07 ± 6.22. BA for those older than 60 years was 68.14 ± 9.63 and δ-age was −0.39 ± 6.63. BA (KDM) for those less than or equal to 40 years of age was 22.46 ± 8.97, with a δ-age of −6.65 ± 8.61. BA for those aged 40–60 years was 36.53 ± 11.19, and δ-age was −12.72 ± 10.57. BA was 47.76 ± 12.90 for those >60 years old, and δ-age was −20.76 ± 12.87. Both BA strata show the greatest difference between BA and CA for those >60 years old, the older you get the more likely you are to experience physical aging. This suggests a heightened need to focus on the psychological age of the older adult population.

Health behavior indicators, including physical activity, smoking, and alcohol consumption, influence both BA and δ-age. For instance, smoking, heavy drinking, and vigorous physical activity correlate with an increased whole-body δ-age. In the population aged over 60, changes in δ-age were most significantly influenced by smoking, heavy alcohol consumption, and high levels of physical activity (P < 0.01). Diabetes mellitus and hypertension significantly affect individuals under 40 years of age (P < 0.01). Participants with chronic conditions, including hypertension and diabetes, exhibited comparable trends in both BA and δ-age.

### 3.3 The relationship between depressed mood and BA

Primarily, in the linear analysis (Table 2), model 1 corrects for demographic variables (gender, age, race, education, income). Model 2 further corrects for BMI, PA, smoking, and alcohol consumption based on model 1. Ultimately, model 3 corrects for diabetes and hypertension on top of model 2. In the unweighted model, Model3 results showed a positive association between PHQ-9 scale (depression scale) scores and accelerated aging (PHE-advance) (β = 0.066,95% CI=(1.039,1.098),P < 0.001). In a weighted model, a stable positive association was found between PHQ-9 scale (depression scale) scores and accelerated aging (PHE-advance) (β = 0.087,95% CI=(1.051,1.132),P < 0.001). Linear regression indicates a positive correlation between depressive mood and accelerated aging. Meanwhile.

**TABLE 2:** Linear association between PHQ-9 scale (depression scale) scores and accelerated aging.

Variable	β (95%CI) without weighted simple			β (95%CI) weighted simple		
	Model 1	Model 2	Model 3	Model 1	Model 2	Model 3
PHE-advance						
Depression scores	0.123 (1.096,1.167)^***^	0.086 (1.058,1.122)^***^	0.066 (1.039,1.098)^***^	0.141 (1.112,1.193)^***^	0.112 (1.078,1.161)^***^	0.087 (1.051,1.132)^***^
KDM-advance						
Depression scores	0.016 (0.962,1.073)	0.011 (0.953,1.072)	0.029 (0.971,1.092)	0.059 (0.996,1.129)	0.029 (0.976,1.085)	0.001 (0.948,1.057)

^*^ P<0.05,^**^ P < 0.01,^***^P < 0.001.

OR, odds ratio; CI, confidence intervals.

Model 1: gender + age + edu + race + income.

Model 2: Model 1+BMI + PA + smoke + drink.

Model 3: Model 2+hypertension + diabetes.

Secondarily, in the logistic regression of the unweighted model (Table 3), the results showed that the presence of depressed mood was associated with a higher risk of aging (OLD-PHE) (OR = 1.183, 95% CI=(1.020,1.371),P = 0.026). Similarly, the presence of depressed mood was associated with a higher risk of aging (OLD-PHE) in the logistic regression of the weighted model (OR = 1.021, 95% CI (1.009,1.032), P = 0.019). Therefore, depressive mood emerged as an independent modifiable factor contributing to accelerated aging trajectories, even after adjusting for lifestyle confounders.

**TABLE 3:** Logistic regression between PHQ-9 scale (depression scale) scores and accelerated aging.

Variable	OR (95%CI) without weighted simple			OR (95%CI) weighted simple		
	Model 1	Model 2	Model 3	Model 1	Model 2	Model 3
OLD-PHE						
Depression scores	1.035 (1.024,1.046)^***^	1.025 (1.015,1.037)^***^	1.020 (1.009,1.032)^***^	1.020 (1.025,1.053)^***^	1.039 (1.017,1.049)^***^	1.020 (1.009,1.042)^***^
Depression(no)						
yes	1.388 (1.210,1.594)^***^	1.255 (1.087,1.447)^**^	1.183 (1.020,1.371)^*^	1.508 (1.024,1.046)^***^	1.409 (1.015,1.037)^**^	1.027 (1.009,1.032)^*^
OLD-KDM						
Depression scores	1.006 (0.993,1.019)	1.000 (0.986,1.013)	1.000 (0.987,1.014)	1.008 (0.991,1.024)	1.001 (0.985,1.018)	0.995 (0.976,1.013)
Depression(no)						
yes	1.059 (0.888,1.258)	0.995 (0.832,1.184)	1.001 (0.836,1.194)	0.938 (0.749,1.174)	0.859 (0.680,1.085)	0.787 (0.615,1.006)

^*^ P<0.05,^**^ P < 0.01,^***^P < 0.001.

OR, odds ratio; CI, confidence intervals.

Model 1: gender + age + edu + race + income.

Model 2: Model 1+BMI + PA + smoke + drink.

Model 3: Model 2+hypertension + diabetes.

Finally, in the logistic regression of the unweighted model, the results showed an association between the presence of a heavier depressive mood and a higher risk of aging (OLD-PHE) (OR = 1.020, 95% CI = (1.009,1.032), P < 0.001). Similarly, in the logistic regression of the weighted model, there was an association between heavier depressive mood and higher risk of aging (OLD-PHE), (OR = 1.025, 95% CI (1.009,1.042), P = 0.003). Convergently, a significant correlation was observed between the severity of depressive mood and the risk of accelerated aging.

Taken together, these findings position depression as a modifiable accelerator of geroprotective pathway dysregulation. Early intervention in depressive disorders could attenuate BA, suggesting novel targets for longevity therapeutics.

### 3.4 Dose-response relationship of the depression scale PHQ-9 scores and risk of aging

**FIGURE 2:**
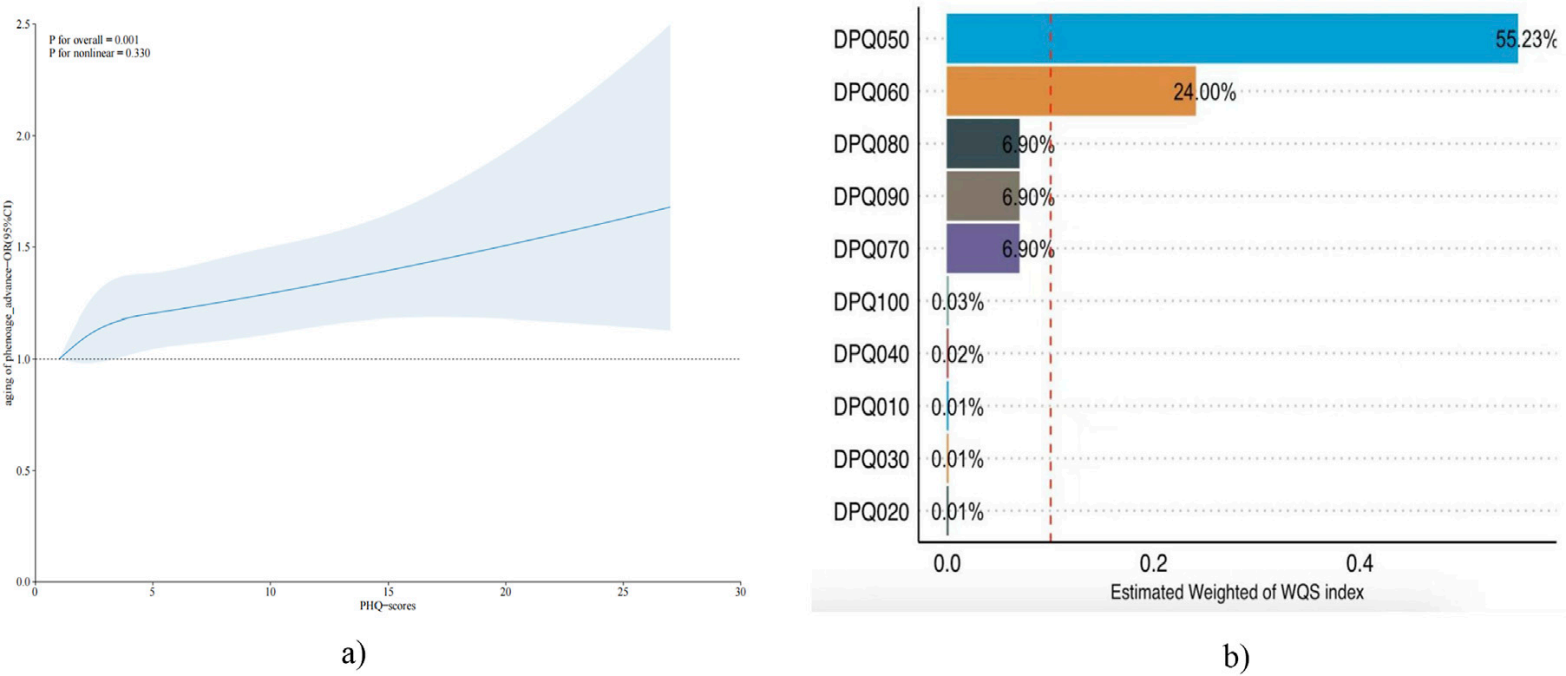
**(a)** The RCS test chart for aging of phenoage_advance and PHQ-scores. **(b)** Estimated Weight of WQS index.

To further validate the relationship between depression and aging, the RCS to validate a dose-response relationship between continuous depression scores and risk of aging was used Figure 2 illustrates the positive dose-response relationship between depression scores and biological survival of aging (OLD-PHE) in the positive (P = 0.001), and there is no non-linear relationship (P non-linear = 0.330).

### 3.5 Mixed effects model of PHQ-9 scores in the risk of aging

To further validate and explore the association of depression and aging risk. The WQS model was used to examine the mixed effects and weight sizes of depression scale components in the risk of aging. WQS modelling results show a positive trend in depression scores and risk of biological aging, but not statistically different (Table 4). Among the biggest contributors to biological aging are too little or too much appetite and low self-esteem (Figure 2). Both kdm and phenoage are indicators of BA. KDM has been previously associated with depression in the literature. However, no significant association was detected between KDM and depression in the present study (Gao et al., 2023).

**TABLE 4:** Mixed-action relationship between depressive components and biological aging.

Variable	OR (95%CI)			*P*
	OR	2.5%	97.5%	
Old-phe				
Model 1	1.218	0.993	1.493	0.058
Model 2	1.189	0.951	1.489	0.129
Model 3	1.358	0.998	1.849	0.052
Old-kdm				
Model 1	1.203	0.691	2.094	0.515
Model 2	0.986	0.58	1.676	0.959
Model 3	0.877	0.519	1.482	0.625

OR, odds ratio; CI, confidence intervals.

Model 1: gender + age + edu + race + income.

Model 2: Model 1+BMI + PA + smoke + drink.

Model 3: Model 2+hypertension + diabetes.

## 4 Discussion

Aging populations exert growing pressure on global public health systems. Therefore, decelerating the aging process through dietary and lifestyle modifications is crucial. In this cross-sectional study with a design of 7,383 participants, depressive mood correlates with biological age, aligning with Lorenzo’s study (Lorenzo et al., 2023). Several studies have shown that depressed mood is strongly associated with aging and frailty (Soysal et al., 2017). However, a paucity of research exists that directly correlates depressed mood with BA. Depressive moods are debilitating, a condition congruent with the symptoms of aging. So the hypothesis that depressive moods may accelerate aging was tested, and the findings from this study support this notion. Extensive research indicates that models of depression frequently incorporate stress-induced behavioral changes. Stress activates various hypothalamic regions and enhances the secretion of pro-inflammatory cytokines (Li et al., 2017). Additionally, stress disrupts the equilibrium of the hypothalamic-pituitary-adrenal (HPA) axis (Pedraz-Petrozzi et al., 2020). The association between immune inflammation activation and major depression was first identified in the 1990s, including increases in IL-18, etc. The first relevant clinical evidence comes from the study of Maes M (Maes et al., 1992). IL-1 rises with fatigue and somatic symptoms such as insomnia and pain hypersensitivity (Maes et al., 2012) and in the context of chronic fatigue syndrome, IL-1 is a significant factor (Roerink et al., 2017). Major depression is strongly correlated with chronic fatigue syndrome (Chaves-Filho et al., 2019). TNF-α, positively linked to depression, concurrently elevates somatic fatigue (Pedraz-Petrozzi et al., 2020). In patients with major depression, diminished antioxidant levels in blood augment inflammatory pathway activity and escalate inflammatory mediators, ultimately leading to neuronal apoptosis (Bhatt et al., 2020).

Sleep disturbances are a significant clinical feature of depression (MORPHY et al., 2007)and also worsen depression (Morehouse et al., 2002). Studies using rats showed that continued REM sleep deprivation induced depressive-like behavior (Ma et al., 2019). Sleep disturbances disrupt the hypothalamic-pituitary-adrenal (HPA) axis (de Leeuw et al., 2023) and activate nuclear factor‐kappaB (NF‐κB), a key transcriptional control pathway in the inflammatory signalling cascade, then increase IL‐6 and TNF (MILLER et al., 2009) Finally, depressive mood and sleep deficiency can worsen fatigue (Sunwoo et al., 2022).

Patients with depression frequently exhibit changes in eating behaviors and consumption, overeating-induced obesity disrupts serotonin synthesis. A deficiency in serotonin is a potentially significant contributor to depression (Markowitz et al., 2008) High-fat diets can induce mood changes via alterations in gut microbiota (Van Oudenhove et al., 2011). Diet and emotional stress can alter mood and elevate inflammatory factors (Freeman and Rapaport, 2011). Therefore, an unhealthy diet can lead to an increase in aging-associated microbiota, as well as a higher risk of age-related cardiovascular diseases and degenerative diseases (Sanchez-Morate et al., 2020). Particularly, long-term consumption of pro-inflammatory foods, such as high-sugar diets, refined grains, and fried foods, can exacerbate inflammation and ultimately lead to frailty (Jalili et al., 2023).

In summary, on the one hand, depression is associated with changes in inflammatory markers and endocrine dysfunction. Studies have shown that depression is linked to elevated levels of pro-inflammatory cytokines such as IL-6, TNF-α, and CRP. Additionally, depression is often accompanied by dysfunction of the hypothalamic-pituitary-adrenal (HPA) axis, which can lead to increased cortisol levels and further exacerbate inflammatory responses. These biological changes not only contribute to the pathophysiology of depression but also have a significant impact on overall health and aging. On the other hand, depression affects income, education, and social interactions, reduces physical activity and health-promoting behaviors associated with healthy aging, and further accelerates the aging process (Crimmins, 2020). In contrast, positive emotions tend to promote healthier lifestyle practices, thereby contributing to the deceleration of the aging process (Chida and Steptoe, 2008), and positive emotions correlate with enhanced cognitive and neurobiological profiles, potentially contributing to slowed aging (Cotter et al., 2020).

In view of this, heightened attention should be given to the mental health of the general population. Positive mental adjustment, regular sleep, and a balanced diet and exercise regimen can decelerate the aging process. Based on the relevant research and our analysis, we revealed the strongest correlations between changes in appetite and δ-age, decreased self-evaluation ranked as the second most notable impact of age-related changes. The paradoxical rise in life expectancy has amplified socioeconomic burdens associated with population aging, underscoring the urgent need to elucidate modifiable determinants of aging trajectories and develop deceleration strategies. However, chronological age fails to capture interindividual heterogeneity in biological aging processes. In contrast, biological aging metrics not only integrate multifactorial influences encompassing environmental exposures, genetic predispositions, lifestyle choices, and psychosocial stress profiles, but also enable cost-effective quantification through validated methodologies. Systematic analysis of biological aging signatures allows identification of pro-aging factors amenable to targeted interventions, thereby facilitating precision strategies for aging rate modulation. This investigation marks the first to establish a dose-response association between depressive symptoms and BA. Depression is associated with accelerated aging through multiple mechanisms.

This study offers several advantages over prior research findings. Firstly, the effect of depressed mood on whole-body aging was assessed using BA as an outcome variable. Secondly, our research indicates that a positive mental attitude significantly slows aging processes. Lastly, this study leverages a large population-based survey, NHANES, which utilizes a stringent random sampling methodology to ensure the representativeness of our findings across the entire demographic spectrum. However, it is important to acknowledge the limitations inherent in our study. Firstly, it used a cross-sectional study design that precluded the establishment of a causal or temporal association between depressed mood and whole-body aging. Secondly, there were inaccuracies in the assessment based on depressed mood, although we excluded participants who were unable to be interviewed. Finally BA was measured by several blood biomarkers that may not accurately represent other assessments of BA, such as brain age, telomere length and DNA methylation. However, these markers are costly and not widely accessible. Additionally, while the National Health and Nutrition Examination Survey (NHANES) is a highly representative database, we acknowledge the limitations inherent in analyzing a single dataset. In our future research, we will address this limitation by exploring the relationship between depression and aging across multiple cohorts and diverse populations. This approach will allow us to validate our findings and enhance the generalizability of our conclusions. We will ensure that our subsequent studies incorporate a broader range of datasets to provide a more comprehensive understanding of the topic.

## 5 Conclusion

This NHANES cohort study indicates that a depressed mood is associated with systemic BA and is the first population-based study to use blood-based biomarkers to assess BA and examine the link between depressive mood-like and whole-body aging. Notably, irregular dietary patterns exert the most significant impact on whole-body BA. These findings underscore the necessity of heightened attention to public mental health, advocating for strategies to mitigate aging and enhance holistic wellbeing, including through dietary and emotional modulation.
